# The median effective concentration (EC50) of propofol with different doses of fentanyl during colonoscopy in elderly patients

**DOI:** 10.1186/s12871-016-0189-y

**Published:** 2016-04-21

**Authors:** Shiyang Li, Fang Yu, Huichen Zhu, Yuting Yang, Liqun Yang, Jianfeng Lian

**Affiliations:** 1 Department of Anesthesiology, Quanzhou Children’s Hospital, Fujian Medical University, Fujian, 362000 China; 2 Department of Anesthesiology, Renji Hospital, Shanghai Jiaotong University School of Medicine, Shanghai, 200136 China

**Keywords:** EC50, Propofol, Fentanyl, Elderly patients

## Abstract

**Background:**

Propofol and fentanyl are the most widely administered anesthesia maintaining drugs during colonoscopy. In this study, we determined the median effective concentration (EC50) of propofol required for colonoscopy in elderly patients, and the purpose of this study was to describe the pharmacodynamic interaction between fentanyl and propofol when used in combination for colonoscopy in elderly patients.

**Methods:**

Ninety elderly patients scheduled for colonoscopy were allocated into three groups in a randomized, double-blinded manner as below, F0.5 group (0.5 μg.kg^−1^ fentanyl), F1.0 group (1.0 μg.kg^−1^ fentanyl) and saline control group. Anaesthesia was achieved by target-controlled infusion of propofol (Marsh model, with an initial plasma concentration of 2.0 μg.ml^−1^) and fentanyl. Colonoscopy was started 3 min after the injection of fentanyl. The EC50 of propofol for colonoscopy with different doses of fentanyl was measured by using an up-and-down sequential method with an adjacent concentration gradient at 0.5 μg.ml^−1^ to inhibit purposeful movements. Anaesthesia associated adverse events and recovery characters were also recorded.

**Results:**

The EC50 of propofol for colonoscopy in elderly patients were 2.75 μg.ml^−1^ (95 % CI, 2.50–3.02 μg.ml^−1^) in F0.5 group, 2.05 μg.ml^−1^ (95 % CI, 1.98–2.13 μg.ml^−1^) in F1.0 group and 3.08 μg.ml^−1^ (95 % CI, 2.78–3.42 μg.ml^−1^) in control group respectively (*P* < 0.05). Patients in the F1.0 group had a significantly longer awake time and length of hospital stay than those in control group (*P* < 0.05).

**Conclusion:**

Increasing doses of fentanyl up to 1.0 μg.kg^−1^ reduces the propofol EC50 required for elderly patients undergoing colonoscopy, and there was no significant difference in anaesthesia associated adverse events but prolonged awake and discharge time.

**Trial registration:**

Chinese Clinical Trial Registry ChiCTR15006368. Date of registration: May 3, 2015.

## Background

Colonoscopy is an essential screening and therapeutic tool for colorectal diseases. It is usually performed under sedation for higher patient acceptance and comfort [1, 2]. Propofol is a sedative-hypnotic drug and characterized by a rapid onset and shorter recovery time with less postoperative nausea and vomiting [3, 4]. As a result, propofol is appropriate for diagnostic and therapeutic procedures such as outpatient anaesthesia procedures, including digestive endoscopy [5], especially with a target controlled infusion pattern [6]. Adjuvants are usually needed in many cases because such co-administration can improve anaesthetic efficacy as well as patients’ satisfaction [7]. Fentanyl is a short-acting potent opioid and is the most used opiate during colonoscopy [2]. Whereas, the co-administration of such drugs could induce supress of airway reflex, thus increasing the risk of desaturation during colonoscopic procedures. Elderly patients are the most sensitive population to hypoxemia. Because they are usually complicated with cardiopulmonary diseases and impaired renal or hepatic function [8]. Since colonoscopy is usually carried out in ambulatory settings, such contradiction could prolong the recovery time and complicate the management [9, 10]. So, it is crucial to determine the pharmacodynamic interaction of propofol and fentanyl during sedation procedures in elderly patients. We therefore designed this randomized, double-blinded and controlled study to characterizing the effect of different doses of fentanyl on the median effective concentration (EC50) of propofol for elective colonoscopy in elderly patients, which we hope could provide new insights to a better management for such clinical situations.

## Methods

### Study design and patient population

This randomized, double-blinded and controlled study was approved by the Ethics Committee of Shanghai Renji hospital and was also registered in Chinese Clinical Trial Registry ChiCTR15006368. Elderly patients from the outpatient department for colonoscopy from November, 2013 to July, 2014 were enrolled in the study and patients’ consents were signed before the surgical procedure.

### Criteria for inclusion and exclusion

Patients were included if they (1) were 75–89 years old; (2) were with American Society Anesthesiologists physical status (ASA) I–III; (3) had body mass index (BMI) 19–27 k.m^−2^.

Patients were excluded when they had (1) allergy to either propofol or fentanyl; (2) chronic use of analgesics; (3) alcohol abuse; (4) neurological or pyschiatric disorders; (5) end stage liver or renal diseases or (6) sleep Apnea Hypopnea Syndrome (SAHS).

### Preoperative preparations and anesthesia protocol

During the preoperative evaluation, informed consent was signed by the patient and his/her family member. Patients were divided into one of this three groups by a random number generated by computer, F0.5 group (0.5 μg.kg^−1^fentanyl, *n* = 30), F1.0 group (1.0 μg.kg^−1^ fentanyl, *n* = 30) and saline control group (equivalent volume of normal saline, *n* = 30).

Upon arrival of the patient in the operating room, an antecubital venous access was established and then Ringer Lactate solution was administered. A nasal oxygen catheter was started with a flow of 3 L.min^−1^. Heart rate (HR), blood pressure (BP), electrocardiogram (ECG) and pulse oximetry (SpO_2_) were monitored continuously during the study.

After preoxygenation, computer-controlled TCI (Graseby™ 3500 TCI Syringe Pump, London UK) was started, propofol (AstraZeneca Company, Italy) was administered with a Marsh pharmacokinetic parameters with an initial plasma target concentration at 2.0 μg.ml^−1^ in this three groups. We started the first dose of propofol at 2.0 μg.ml^−1^since previous study determined that the Cp50 of propofol to make elderly patients population to lose consciousness was within this range. Once the target concentration achieved, fentanyl was injected immediately. All fentanyl solutions were diluted into 5 ml and was prepared by a nurse who was no longer involved in the follow-up. Patients were injected with different doses of fentanyl: 0.5 μg.kg^−1^ fentanyl (F0.5 group), 1.0 μg.kg^−1^ fentanyl (F1.0 group) and blank saline solution (Control group).

Qualified endoscopists started colonoscopy procedure 3 min after fentanyl injection and TCI pump was suspended just before the end of the procedure. Purposeful movements, including head or limbs purposeful movements during colonoscopy was defined as “responsive”. If the patient presented “responsive”, the target plasma concentration of propofol for the next patient would be augmented with an adjacent concentration gradient at 0.5 μg.ml^−1^. If there was no purposeful movements occurred, which defined as “non-responsive”, a lower-level concentration which was decreased with an adjacent gradient at 0.5 μg.ml^−1^would be adopted for the next case. This method was called up-and-down method of Dixon, described as early in 1965 [11].

Hypotension, which was define as a BP decrease of 30 % over the baseline value, was treated by fluid therapy with accelerated infusion of Ringer Lactate and bolus of 6–10 mg ephedrine. Bradycardia (HR < 50 bpm) was treated with bolus of 0.5 mg atropine. Respiratory depression (SpO_2_ less than 90 %) refractory to continuous oxygen (3 L.min^−1^) inhalation was corrected by unarmed airway opening. If pulse oximetry not improved or spontaneous respiration always absent, an artificial ventilation support would be provided.

### Measurements

Baseline BP and HR were defined as the mean of the two lowest measurements recorded during 3 min interval just before the start of anesthesia. Values from all routine anaesthetic monitors were recorded at a 3-min interval duration of surgical procedure, while perioperative anaesthesia related adverse events, such as hypotension, bradycardia and frequency of respiratory depression also recorded. The pump infusion of propofol was stopped at the end of colonoscopy, which was defined as the removal of the colonoscope. The time from discontinuing propofol to obeying commands of eye opening was defined as awake time. During postoperative PACU stay, the occurrence of postoperative nausea and vomiting (PONV) were recorded and treated with antiemetics. The criteria for discharging patients from the endoscopy unit were listed as following: ability to respond appropriately to questions, sitting upright for at least 5 min, and dressing without assistance. The time from eye opening to discharge was recorded as hospital discharge time.

### Statistical analysis

Studies have showed that anaesthesia studies using the up-and down method typically need 20–40 patients per group [4, 12]. We have also anticipated a lost follow-up incidence of about 10 %. Therefore, the sample size was set at 30 patients for each group. The EC50 of propofol in each group was determined by a modification of Dixon’s up-and-down method [4] and defined as the mean cross-over midpoint in each group. Statistical analysis were performed by ANOVA and the comparisons among EC50 groups depend on Mann-Whitney *U* test. Values are expressed as mean and standard deviation or number of patients. A *P* value less than0.05 was considered to be statistically significant.

## Results

All 90 patients completed the study without incorrigible adverse effects such as severe hypoxemia, bradycardia or hypotension. Patients’ demographic data and characteristics are presented in Table 1, and they were similar in terms of age, weight, BMI, ASA physical status, coexisted diseases and colonoscopy duration (*P* > 0.05).

**Table 1:** Demographic data and patients’ characters (*n* = 30 in each group)

	Control	Group F0.5	Group F1.0
Gender (M/F)	17/13	14/16	15/15
Age (yrs)	78.2 ± 9.2	79.6 ± 6.1	82.1 ± 7.9
BMI (kg/m^2^)	23.3 ± 2.2	25.2 ± 1.5	24.5 ± 2.5
ASA (II/III)	24/6	25/5	27/3
Hypertension	26/4	24/6	26/4
Diabetes	17/13	18/12	15/15
Drug allergies	3/27	3/27	4/26
Smoking history	11/19	12/18	11/19
Snoring	10/20	9/21	8/22
Colonoscopy time (min)	15.2 ± 1.8	14.6 ± 2.2	16.2 ± 1.9

Values are expressed as mean ± SD or number of patients

**Fig. 1:**
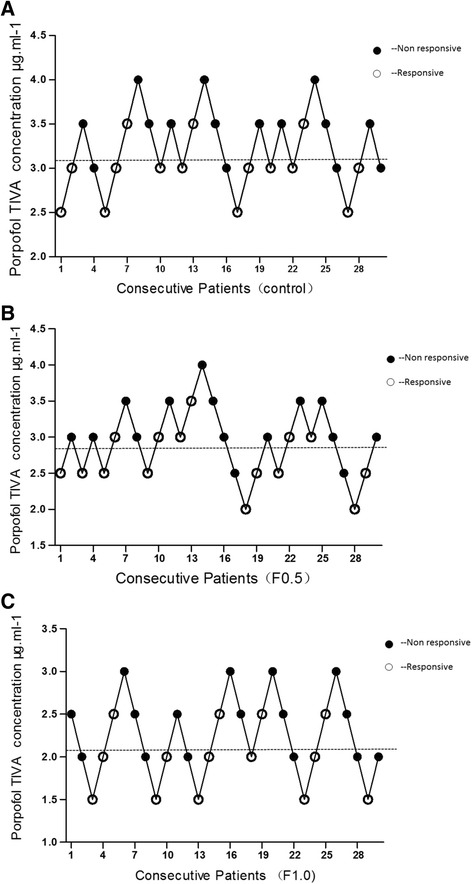
Consecutive target propofol concentrations during colonoscopy insertion for EC50 determination. The lines represent the mean propofol concentration when crossing from a failure to suppress patient movements. The average of these concentrations is defined as EC50. Propofol EC50 was 3.08 with 95 % CI of 2.78–3.42 μg.ml^−1^ in control group (shown in Fig. 1**a**); while EC50 in F0.5 group was 2.75 with 95 % CI of 2.50–3.02 μg.ml^−1^ (shown in Fig. 1**b**) and 2.05 with 95 % CI of 1.98–2.13 μg.ml^−1^ in F1.0 group (shown in Fig. 1**c**)

The target concentration of propofol in plasma for consecutive patients and their responses to the colonoscopy procedure are shown in Fig. 1. Propofol EC50 and its 95 % confidence interval were 2.75 (2.50–3.02) μg.ml^−1^ in F0.5 group, 2.05 (1.98–2.13) μg.ml^−1^in F1.0group and 3.08 (2.78–3.42) μg.ml^−1^ in Control group respectively (shown in Table 2). When EC50 of propofol compared among groups, there was no statistically significant difference between F0.5 group and Control group (*P* > 0.05), whereas, EC50 of propofol in F1.0 group was statistically significantly decreased compared to Control group (*P* < 0.05).

**Table 2:** Propofol EC50, anesthesia related adverse events and recovery time (*n* = 30 in each)

	Control	Group F0.5	group F1.0
Propofol EC50 (μg.ml^−1^)	3.08	2.75	2.05^*^
95 % CI (μg.ml^−1^)	2.78–3.42	2.50–3.02	1.98–2.13
Adverse events			
Hypotension	2	1	1
Bradycardia	2	3	1
Respiratory depression	1	1	2
PONV	2	3	6 ^*^
Total	7 (23.3 %)	8 (26.7 %)	10 (33.3 %)
Awake time (min)	6.2 ± 2.4	7.8 ± 3.1	9.6 ± 3.8^*^
Hospital discharge (min)	35.7 ± 5.9	38.2 ± 7.5	47.6 ± 10.2^*^

Values are expressed as mean ± SD or number of patients

^*^ *P* < 0.05

The incidence of adverse events during colonoscopy were listed in Table 2. There were no significant difference in total anaesthesia related adverse events among three groups, while the incidence of PONV was significantly increased in F1.0 group. Patients in F1.0 group but not F0.5group had statistically significantly longer awake time and length of hospital stay (*P* < 0.05, Table 2).

## Discussion

The primary objective of this study was to evaluate the impact of the combination of fentanyl on the needs of propofol to achieve a targeted sedative depth for colonoscopy in elderly patients. Our results show a significant reduction in propofol requirement when co-administration with 1 μg.kg^−1^of fentanyl rather than 0.5 μg.kg^−1^. This finding was meaningful because it confirmed that anaesthetics had interactions and an optimal combination method should be investigated to ensure both safety and effectiveness, especially for elderly patients. Because many studies demonstrated that the propofol requirements are reduced in elderly individuals [13, 14]. This can be explained by age-related changes in pharmacokinetics, pharmacodynamics or both [15]. Other study confirmed that age increases the sensitivity of the brain to propofol [14].

Our study has adopted a plasma TCI infusion mode for propofol. Studies have confirmed that the onset and recovery time of propofol by plasma TCI mode were significantly shorter than traditional infusion mode [9, 10]. Fentanyl is frequently used with propofol for sedative surgical procedures and the usual doses are usually set at 50–200 micrograms [1]. We administrated patients with fentanyl at 0.5 μg.kg^−1^ or 1.0 μg.kg^−1^, which is perfectly located in the safe dosage spectrum.

Sequential design methods are usually employed for the determination of the concentration or dose associated with the 50 % point along the dose-response curve, so EC50 or ED50 is commonly used as a measure of drug’s potency [16, 17]. The up-and down method of Dixon has a very long history [18, 19] and is commonly used in anesthesia research [4]. Now, developments in statistical methods have allowed study designs for the measurements of the response at any point along the dose-response curve. Since propofol and fentanyl have “synergic effects”, their interactions should be further investigated to make sure a both safe and potent anaesthesia for all patients.

Several limitations in the present study should be mentioned. Firstly, we defined purposeful head or limbs movement during surgical procedure as “responsive”, however, it is subjective and further studies combined with quantified methods, such as BIS or entropy monitoring are highly needed [20]. Secondly, we applied Marsh TCI model for propofol infusion. Studies showed that Marsh model does not make any adjustments for age [21], since it assumes identical concentrations of the drugs, regardless of the age. So, the concentration in our study might actually be higher than expected and lead to hemodynamic instability [20, 21]. Other models, taking age factor into account, such as Schnider model [21] should be considered in our next step. Finally, due to the long duration of the study, large number of anaesthesiologists and nurses were involved, and thus, the results were slightly inclined to bias.

## Conclusions

In conclusion, this randomized, double-blinded and controlled trial for elderly patients undergoing ambulatory colonoscopy showed that EC50 of propofol detected by Dixon’s up-and-down method in such procedure was decreased when combined with 1 μg.kg^−1^ fentanyl. No severe adverse events were observed in present study except prolonged recovery time after anaesthesia.
